# Monoclonal Gammopathy of Undetermined Cardiovascular Significance; Current Evidence and Novel Insights

**DOI:** 10.3390/jcdd10120484

**Published:** 2023-12-04

**Authors:** Anastasios Tentolouris, Ioannis Ntanasis-Stathopoulos, Maria Gavriatopoulou, Ioanna Andreadou, Evangelos Terpos

**Affiliations:** 1 First Department of Propaedeutic Internal Medicine and Diabetes Center, School of Medicine, National and Kapodistrian University of Athens, Laiko General Hospital, 11527 Athens, Greece; antentolouris@hotmail.com; 2 Department of Clinical Therapeutics, School of Medicine, National and Kapodistrian University of Athens, Alexandra General Hospital, 11528 Athens, Greece; 3 Laboratory of Pharmacology, Faculty of Pharmacy, National and Kapodistrian University of Athens, 15771 Athens, Greece

**Keywords:** MGUS, cardiovascular disease, myocardial infarction, stroke, peripheral arterial disease

## Abstract

Monoclonal gammopathy of undetermined significance (MGUS) is a premalignant condition characterized by the presence of low levels of a monoclonal protein in the serum and a low percentage of clonal plasma cells in the bone marrow. MGUS may progress to multiple myeloma or other plasma cell disorders at a rate of 1% annually. However, MGUS may also have adverse effects on the cardiovascular system independent of its malignant potential. Emerging data have shown that MGUS is associated with cardiovascular disease. The mechanisms underlying this association are not fully understood but may involve genetic abnormalities, vascular calcification, cryoglobulinemia, cold agglutinin disease, autoantibodies and the direct or indirect effects of the monoclonal protein on the vascular endothelium. Herein, we review current evidence in this field and we suggest that patients with MGUS may benefit from regular cardiovascular risk assessment to prevent severe cardiovascular complications, in parallel with close hematological follow-up to monitor potential disease progression.

## 1. Introduction

Monoclonal gammopathy of undetermined significance (MGUS) is an asymptomatic premalignant plasma cell disorder characterized by the presence of a serum monoclonal protein at a concentration of less than 3 g/dL, bone marrow infiltration by monoclonal plasma cells less than 10% and the absence of end-organ damage (lytic bone lesions, anemia, hypercalcemia, renal insufficiency and hyperviscosity) [1]. Over 3% of people over the age of 50 are diagnosed with MGUS, which is often discovered incidentally when people undergo protein electrophoresis as part of an evaluation for a wide range of clinical symptoms and diseases [1]. The median age at diagnosis is 65–70 years [1], whereas patients with MGUS may have inferior survival compared to the general population [2]. The annual average risk of progression from MGUS to multiple myeloma is 1%, and the major cause of morbidity and mortality in patients with MGUS is progression to multiple myeloma [1].

Although MGUS does not require therapeutic interventions, many people develop unique manifestations related to the monoclonal protein per se in the absence of overt malignancy and are termed as having monoclonal gammopathy of clinical significance [3,4]. Monoclonal gammopathy of renal significance (MGRS) is an example characterized by any B-cell or plasma cell clonal lymphoproliferation with both (a) one or more kidney lesions that are related to the produced monoclonal immunoglobulin and (b) the underlying B-cell or plasma cell clone that does not cause tumor complications or meet any hematological criteria for specific therapy [5,6].

Cardiovascular disease (CVD) consists mainly of ischemic heart disease, cerebrovascular disease, peripheral arterial disease (PAD), heart failure and several other cardiac and vascular conditions. CVD is the leading cause of global mortality, a major contributor to disability, and increased healthcare costs [7]. Patients with multiple myeloma have an increased risk of CVD and recommendations regarding the management of cardiovascular risk have been published [8]. Emerging data have shown that individuals with MGUS may have an increased risk of arterial or venous thrombosis [9]. Recent studies have also shown that individuals with MGUS may have an increased risk of CVD [10,11], and in parallel to the term “MGRS”, the term “monoclonal gammopathy of thrombotic significance” has been proposed [9].

The aim of this narrative review is to examine whether CVD is increased in patients with MGUS, whether MGUS affects CVD outcomes and to investigate the pathophysiologic mechanisms linking MGUS with CVD.

## 2. MGUS and CVD

### 2.1. Cardiovascular Risk Factors in Patients with MGUS

Diabetes mellitus (DM), arterial hypertension, cigarette smoking, high body mass index (BMI), dyslipidemia, chronic kidney disease, low physical activity and nutrition habits are the main modifiable risk factors of CVD [12]. The median age at diagnosis of MGUS is 65 to 70 years; therefore, established CVD risk factors are common in this population. However, data regarding the association between MGUS and CVD risk factors are rather limited.

#### 2.1.1. Diabetes Mellitus

According to the International Diabetes Federation, the global prevalence of DM is 10.5% [13]. The prevalence of DM has not been reported in most of the MGUS studies. Data from studies that have examined whether MGUS is associated with increased CVD risk have reported an elevated prevalence of DM in comparison with the general population [10,14,15]; this might be attributed to the fact that patients with DM who have a high CVD risk may have been recruited in these studies. For example, in a study by Hamadi et al., among 9007 people with MGUS, 3340 (37.1%) had a history of DM [15]. Similarly, Kang et al. reported that, among 470 patients with MGUS, 191 (40.6%) had a history of DM [14]. Schwartz et al. showed that at baseline, among 8189 patients with MGUS, 1064 (13%) had a history of DM, while in the control group, 7586 (9.3%) had a history of DM [10].

Although DM might be associated with the development of MM [16] and DM may be an adverse prognostic factor for overall survival in patients with MM [17], data linking DM and MGUS are limited, with conflicting conclusions due to the overlapping effect of obesity [18]. A population-based matched case-control study in Sweden, with 94,579 cases and 368,348 controls, aimed to examine the association between DM and plasma cell dyscrasias as well as lymphoproliferative disorders [19]. In the univariate analysis, DM was associated with an increased risk of MGUS. However, after controlling for medical visits, DM was not associated with MGUS (Odds Ratio (OR) (95% CI): 0.99 (0.92–1.07)) or with MGUS progression, indicating that detection bias during medical follow-up likely explains the association that was initially observed [19]. Similarly, another study included 2363 patients with MGUS and 9193 matched controls [20]. In the primary analysis, DM was associated with a higher risk of MGUS (OR (95% CI): 1.30 (1.13–1.50)). Nevertheless, after adjustment to the number of laboratory tests prior to the MGUS diagnosis, there was no association between DM and MGUS risk (OR (95% CI): 1.08 (0.93–1.25)) [20]. In addition, among patients with DM, no association was observed between DM duration or glucose levels and MGUS risk. Hence, the probability of detecting an asymptomatic disorder, such as MGUS, is increased in people with medical comorbidities who may require more frequent laboratory testing than the general population. Landgren et al. also examined the association between DM and MGUS and found no significant association [21]. Interestingly, a recently published case report described a case of hypoglycemia due to insulin-binding paraprotein that resolved following treatment with lenalidomide to deplete the plasma cell clone [22].

Hence, further studies are needed to examine whether the prevalence of DM is increased in patients with MGUS and to assess whether DM is associated with the development of MGUS.

#### 2.1.2. Obesity

Similar to DM, body weight, BMI and the prevalence of overweight/obesity among people with MGUS have not been reported in MGUS studies. However, several studies have examined the effect of obesity on MGUS [21,23,24,25], which was thoroughly described in a previous review [26]. In summary, the data were inconsistent and no safe conclusions could be drawn. Prospective studies are needed to examine this relationship [26].

#### 2.1.3. Cigarette Smoking

Smoking status has not been presented in the baseline characteristics of the studies on MGUS; therefore, its prevalence in this patient population is unknown. However, smoking may be associated with MGUS. Pasqualetti et al. performed a retrospective analysis in Italy to assess whether socioeconomic status, residence, alcohol and tobacco habits, occupation, occupational exposure to toxic substances and chronic antigenic stimulation are associated with increased MGUS risk [27]. Overall, 285 patients with MGUS and 570 sex- and age-matched controls were included in this study. Tobacco smoking was associated with an increased risk of MGUS (OR (95% CI):1.49 (1.03–1.89)). Nevertheless, Pasqualetti et al., in a retrospective study one year later, which included 338 people with MGUS, found that MGUS was associated with heavy smoking (OR (95% CI): 2.22 (1.02–4.86)), but not with light smoking (OR (95% CI): 1.31 (0.95–2.28)) [28]. Light smokers were defined as those who smoked less than 20 cigarettes/day for less than 5 years, less than 20 cigarettes per day for more than 5 years or over 20 cigarettes/day for less than 5 years, and heavy smokers were defined as those who smoked more than 20 cigarettes for more than 5 years. Boursi et al. found that a history of smoking was associated with a 30% increased risk for MGUS when compared with those who never smoked (OR (95% CI): 1.30 (1.18–1.44)) [20].

#### 2.1.4. Arterial Hypertension

The global age-standardized prevalence of hypertension (defined as systolic blood pressure ≥ 140 mmHg, diastolic blood pressure ≥ 90 mmHg and/or current use of antihypertensive medication) has been estimated at 31.1% [29]. In the United States, nearly half of adults have hypertension (48.1%), defined as a systolic blood pressure greater than 130 mmHg, a diastolic blood pressure greater than 80 mmHg or taking medication for hypertension [30].

Epstein et al. reported comorbidities among 429 patients with MGUS and 1287 matched controls in the US [31]. Overall, 325 (76%) patients with MGUS and 874 (68%) controls had a history of hypertension. In accordance with these findings, studies examining CVD in people with MGUS have shown that hypertension is among the most common comorbidities [10,14,31]. In the study by Schwartz et al., among 8189 patients with MGUS, 3933 (48%) had a history of hypertension, while among 81,890 controls, 31,541 (38.5%) had a history of hypertension [10]. Similarly, in a study by Kang et al., among 470 patients with MGUS, 254 (54%) had a history of hypertension. In a study by Hamadi et al. of 9007 people with MGUS, 4.324 (48%) had a history of hypertension [15]. To the best of our knowledge, no studies have examined any potential causal association between arterial hypertension and MGUS.

#### 2.1.5. Dyslipidemia

The global prevalence of increased plasma total cholesterol levels among adults aged ≥25 years has been estimated at approximately 39% [32]. In a study by Kang et al., among 470 people with MGUS, 200 (42.6%) had hyperlipidemia [14], while in the study by Epstein et al., among 429 patients with MGUS and 1287 matched controls, 314 (73%) and 899 (70%) had a history of hyperlipidemia, respectively [31]. In a study by Hamadi et al., 3.801 (42.4%) patients had a history of dyslipidemia [15].

Future research should examine whether the presence of DM, hypertension, overweight/obesity or dyslipidemia may be associated with MGUS or whether comorbid diagnoses are related to the process of diagnosing MGUS. Testing bias may be a factor in the overall increased incidence of comorbidity in people with MGUS, since symptoms from these comorbid diseases may have prompted healthcare professionals to order tests that are also utilized to identify MGUS [31]. Overall, if CVD risk factors are more common in patients with MGUS, this results in increased CVD morbidity and mortality.

#### 2.1.6. Chronic Kidney Disease

Chronic kidney disease is a common feature of multiple myeloma, which may also be associated with an increased risk of CVD [33]. MGRS is a term used to describe people who would otherwise meet the criteria for MGUS but have kidney injury that can be attributed to the underlying monoclonal protein [5,6,34]. The prevalence of MGRS is 0.32% and 0.53% in people older than 50 years and 70 years, respectively, and 10% among people with MGUS [5]. Gozzetti et al. performed a retrospective study to assess the prognostic indicators and treatment outcomes of MGRS [35]. A total of 280 adults with biopsy-proven MGRS were included in this study. Amyloidosis related to MGRS was present in 180 patients, and non-amyloidosis MGRS, including a broad spectrum of renal pathologies, was diagnosed in 100 patients. After a median follow-up of 30 months (range 1–192 months) in the amyloidosis-related MGRS, the most common causes of death were disease progression (29%), infection (20%) and heart failure (16%). In the non-amyloidosis MGRS group, disease progression (42%) and infections (25%) were the most common causes of death. Interestingly, only one case of death in the non-amyloidosis MGRS group was related to a CVD event [35].

### 2.2. Cardiovascular Disease Endpoints

Death from any cause, CV death, myocardial infarction (MI), silent MI, hospitalization for unstable angina, stroke, transient ischemic attack, heart failure event, heart failure hospitalization, percutaneous coronary intervention, peripheral vascular intervention and stent thrombosis are some of the most commonly used CVD endpoints [36]. Major adverse cardiovascular events (MACE) constitute a composite endpoint frequently used in CVD studies. The classical “3-point MACE” is defined as a composite of nonfatal stroke, nonfatal MI and CVD death, while “4-point MACE” is commonly defined as “stroke, CVD death, nonfatal MI and hospitalization for heart failure”.

For diseases in which CVD is the major cause of morbidity and mortality, such as DM, the US Food and Drug Administration and the European Medicines Agency issued guidance to the pharmaceutical industry, setting new guidance for the development of drugs [37]. These trials are called Cardiovascular Outcomes Trials, and their target is the new agent to demonstrate non-inferiority and not superiority in terms of CVD [37].

### 2.3. Clinical CVD in Patients with MGUS

Patients with MM have several cardiovascular comorbidities or risk factors for CVD, and MM is associated with increased CV morbidity and mortality [8]. However, emerging data have shown that MGUS is associated with a higher risk of thrombosis, including venous, arterial and microthrombotic events [9].

A recent prospective study examined the effects of MGUS on several CVD parameters, including heart failure, arrhythmias, acute MI, ischemic stroke and PAD (Table 1) [10]. Participants from the Danish National Patient Registry between 1995 and 2018 were included in this analysis. The identification of MGUS cases was based on International Classification of Disease (ICD) coding. Those with the D472 code were classified in the MGUS category. People with MGUS were matched at a ratio of 1:10, based on age and gender, to subjects from the Danish Central Population Registry. Overall, 8189 individuals (51.2% male; mean age 69.8 ± 11.7 years) with MGUS and 81,890 controls (51.2% male, aged 69.8 ± 11.7 years) were included. Among 8189 people with MGUS, 464 (5.7%) had a history of heart failure, 506 (6.2%) had MI, 448 (5.5%) had ischemic stroke and 334 (4.1%) had a history of PAD. After multivariate adjustment, subjects with MGUS had a 22% increased risk for acute MI (HR (95%CI): 1.22 (95% CI: 1.06–1.40)), 16% increased risk for ischemic stroke (HR (95% CI): 1.16 (1.03–1.30)), 69% increased risk for PAD (HR 95% CI): 1.69 (1.47–1.95) and 55% increased risk for heart failure (HR (95% CI): 1.55 (1.41–1.69)) in comparison with the healthy population [10]. A subgroup analysis within the cohort that included people without type 2 DM, hypertension, prior acute MI and chronic kidney disease (3540 people with MGUS and 45,534 controls) showed that the risk remained increased for heart failure (HR (95% CI): 1.67 (1.46–1.91)), acute MI (HR (95% CI): 1.23 (1.01–1.49)) and PAD (HR (95% CI): 2.17 (1.76–2.67)), similar to the overall analysis. However, no significant association was recorded for ischemic stroke (HR (95% CI): 1.14 (0.97–1.35)) [10].

In a population-based study in Sweden, 5326 MGUS cases were matched to 20,161 controls, and 18,627 multiple myeloma cases were matched to 70,991 controls between 1958 and 2006 to examine the risk of venous and arterial thrombosis in patients with MGUS and multiple myeloma [38]. Coronary atherosclerotic disease (CAD) was defined as angina pectoris, unstable angina and MI; cerebrovascular disease was defined as cerebral infarction, transient ischemic attack and cerebral hemorrhage. After 1, 5, and 10 years of follow-up, subjects with MGUS had 2, 1.5, and 1.5, higher risk for CAD, respectively, than controls (HR (95% CI): 2 (1.7–2.4), 1.5 (1.3–1.7), and 1.5 (1.3–1.6), respectively). In addition to the above, after 1, 5, and 10 years of follow-up, there was a trend for an increased risk of cerebrovascular disease, which was defined as cerebral infarction, transient ischemic attack, and cerebral hemorrhage, in people with MGUS when compared with healthy controls (HR (95% CI): 1.4 (1.0–1.7), HR (95% CI): 1.1 (1.0–1.3), HR (95% CI): 1.1 (1.0–1.3), respectively). Interestingly, in an analysis based on the MGUS isotype, subjects with IgG/IgA MGUS had a significantly increased risk of both venous and arterial thrombosis, while those with IgM MGUS did not have an increased risk of venous or arterial thrombosis compared with controls. In comparison, patients with multiple myeloma had 2.2, 1.8 and 1.7 times increased risk for CAD after 1, 5 and 10-year follow-up, respectively, and 1.5, 1.2 and 1.2 times increased risk for cerebrovascular disease after 1, 5 and 10-year follow-up, respectively. Hence, individuals with MGUS showed a lower risk of CAD and cerebrovascular events than patients with MM and a higher risk when compared with healthy controls. Furthermore, patients with both MM or MGUS had the highest risk of CAD and cerebrovascular disease during the first year following the diagnosis of plasma cell disorder [38].

**Table 1:** Overview of studies evaluating risk of CVD in patients with MGUS.

Study	Study Design	Population	Outcome—Effect Estimate
Hamadi et al., 2023 [15]	Retrospective study	9007 MGUS 2404 MGUS + AF 6603 MGUS without AF	2920 (32.4%) of people with MGUS had CAD 748 (8.3%) of people with MGUS had stroke
Schwartz et al., 2022 [10]	Prospective study	8189 MGUS 81,890 controls	Acute MI infarction (HR (95%CI): 1.22 (95% CI: 1.06–1.40)) Ischemic stroke (HR (95% CI): 1.16 (1.03–1.30)) PAD (HR (95% CI): 1.69 (1.47–1.95))
El Khoury et al., 2022 [39]	Cohort study	592 MGUS 3615 controls	Coronary artery disease MGUS (OR (95% CI): 1.22 (0.97–1.53)) Acute myocardial infarction MGUS (OR (95% CI): 1.39 (1.07–1.80))
Kang et al., 2021 [14]	South Korea nationwide registry-based study	470 MGUS 405 MGUS without comorbidities at the time of MGUS diagnosis	73 (18%) myocardial infarction events 67 (16.1%) stroke events
Kristinsson et al., 2010 [38]	Swedish nationwide retrospective cohort study	5326 MGUS 20,161 controls	Coronary artery disease 1-year follow-up: HR (95% CI): 2.0 (1.7–2.4) 5-year follow-up: HR (95% CI): 1.5 (1.3–1.7) 10-year follow-up: HR (95% CI): 1.5 (1.3–1.6) Cerebrovascular disease 1-year follow-up: HR (95% CI): 1.4 (1.0–1.7) 5-year follow-up: HR (95% CI): 1.1 (1.0–1.3) 10-year follow-up: HR (95% CI): 1.1 (1.0–1.3)

In a 10-year follow-up in South Korea, 470 patients with MGUS were recruited, and the prevalence of comorbidities at the time of MGUS diagnosis as well as the comorbidities that developed during the follow-up period were reported [14]. At the time of MGUS diagnosis, 65 (13.8%) patients had a history of MI, and 55 (11.7%) had a history of stroke. During the 10-year follow-up, among 405 patients without comorbidities at the time of MGUS diagnosis, 73 (18%) were diagnosed with MI, including acute MI, subsequent MI, complications following acute MI, other acute ischemic heart diseases, chronic ischemic heart disease and pulmonary embolism. In parallel, 67 (16.1%) were diagnosed with stroke, a term that included cerebral infarction due to thrombosis of the precerebral arteries, cerebral infarction due to embolism of the cerebral arteries, cerebral infarction due to unspecified occlusion or stenosis of precerebral arteries, cerebral infarction due to thrombosis of cerebral arteries, cerebral infarction due to embolism of cerebral arteries, cerebral infarction due to unspecified occlusion or stenosis of cerebral arteries, cerebral infarction due to non-pyogenic cerebral venous thrombosis and cerebral infarction due to cerebral infarction due to unspecified reasons [14].

In addition, a recent retrospective study aimed to compare the demographic, admission, and medical comorbidity characteristics of patients with MGUS with and without atrial fibrillation (AF) [15]. A total of 9007 patients with MGUS of whom 2404 had atrial fibrillation, were included in the study. Overall, 2920 (32.4%) patients with MGUS had CAD (1093 (45.5%) patients with MGUS and AF had CAD and 1827 (27.7%) patients with MGUS without AF had CAD). Regarding cerebrovascular disease, 748 patients (8.3%) with MGUS had a history of stroke [15].

Another recent multicenter cohort study screened 7622 individuals for the presence of monoclonal gammopathy [39]. The prevalence of MGUS among high-risk individuals (black race or a family history of hematological cancer) 50 years old or above was 13%, evaluated by mass spectrometry. Interestingly, patients with MGUS had a 39% higher risk of AMI (OR (95% CI): 1.39 (1.07–1.80)) and a trend for higher risk of CAD (OR (95% CI): 1.22 (0.97–1.53)) at least 6 months after the time of screening than those without MGUS [39].

In conclusion, MGUS is associated with an increased risk of CVD. Data are more evident for CAD than for cerebrovascular events, whereas data on PAD are limited. Studies examining whether MGUS is associated with subclinical atherosclerosis using markers such as CT calcium score, pulse wave velocity and carotid intima-media thickness are needed to elucidate whether MGUS accelerates atherosclerosis.

### 2.4. MGUS and CVD Outcomes

In addition, MGUS is associated with an increased risk of death due to CVD events. Using population-based and hospital-based registries from Sweden, Kristinsson et al. identified a nationwide cohort of 4259 patients diagnosed between 1986 and 2005 [40]. The causes of death in these patients were compared with those in 16,151 matched controls. The study showed that people with MGUS had an increased risk of dying from CAD (HR (95% CI): 1.3 (1.1–1.4)) and other heart disorders (mainly congestive heart failure, heart valve diseases, cardiomyopathy and arrhythmias) (HR (95% CI): 1.5 (1.2–1.8)) in comparison with healthy controls [40]. In addition, a cohort study examined the outcomes of MGUS in 241 patients between 1956 and 1970 [41]. Among 138 patients with MGUS who died without evidence of symptomatic multiple myeloma, amyloidosis, macroglobulinemia or other lymphoproliferative disease, cardiac disease was the most frequent cause of death (49 patients), followed by cerebrovascular disease (18 patients) [41]. In another study of 1324 patients with MGUS between 1978 and 1993, 868 deaths were reported during 7785 years of follow-up [42]. Malignant transformation was the most common cause of death worldwide. Patients with MGUS had an increased risk of dying from ischemic heart disease (standardized mortality ratio (SMR) (95% CI): 1.6 (1.3–2.0)) in the first 4 years of follow-up; however, no significant association was described for the years 5–18 of the follow-up (SMR (95% CI): 1.2 (0.9–1.5)). There was a trend for increased mortality from cerebrovascular disease in those with MGUS during the years 5–18 of the follow-up (SMR (95% CI): 1.5 (1.0–2.2)) [42]. Similarly, in a long-term follow-up study (median 11.5 years) with 263 cases of MGUS, 157 (59.7%) died of causes unrelated to MGUS; among them, cardiac and cerebrovascular diseases were the most frequent causes of death [43].

To determine whether the presence of MGUS among people with established CVD affects mortality, a retrospective cohort study of 87 patients with CAD and MGUS and 178 patients with CAD without MGUS was performed [44]. The median follow-up period was 2.9 years and the endpoints were the occurrence of MI, stroke, coronary revascularization and all-cause mortality. A total of 60 CV incidents were observed, including 4 cases of MI, 42 cases of coronary revascularization, 12 cases of deaths and 2 cases of stroke. Patients with CAD and MGUS had a higher risk of CVD events than those without MGUS (log-rank *p* = 0.0015).

In conclusion, based on long-term follow-up studies, the primary causes of death in patients with MGUS are malignant transformation and cardiovascular and cerebrovascular disease. Elucidating the underlying pathophysiology is essential to determine a potential causal relationship beyond the probability of co-occurrence as an epiphenomenon.

## 3. Pathophysiological Mechanisms between MGUS and CVD

**Figure 1:**
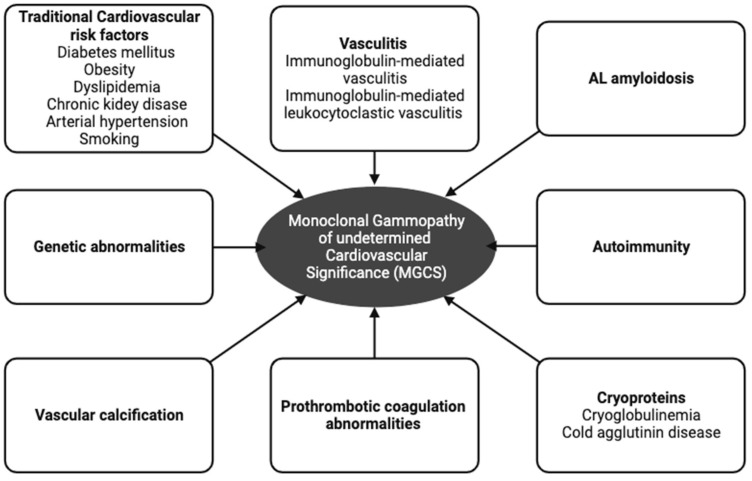
The possible pathophysiological mechanisms between monoclonal gammopathy of undetermined significance and cardiovascular disease.

Overall, data show that patients with MGUS may have an increased risk of CVD events. Although several hypotheses have been proposed, mechanistic studies examining the association between MGUS and CVD are lacking. The possible pathophysiological mechanisms between MGUS and CVD are presented in Figure 1.

### 3.1. Genetic Abnormalities

Emerging data have shown that the pathogenetic mechanisms in hematological malignancies and CVD may have interrelated genetic backgrounds [45]. Clonal hematopoiesis of indeterminate potential (CHIP) is the asymptomatic presence of clones in the bone marrow or peripheral blood that possess somatic gene mutations that are commonly mutated in myeloid neoplasms and have an unpredictable risk of developing into cancer. Carriers of these mutations have a 10-fold increased risk of hematologic malignancy compared to those without such mutations [45]. A study by Testa et al. showed that bone marrow CHIP was detected in approximately 20% of the patients with MGUS and was more frequent among elderly patients [46]. Furthermore, a study by Jaiswal et al. examined whether the presence of genes that lead to CHIP in peripheral blood cells was associated with CAD or early onset MI [45]. A total of 4726 participants with CAD and 3529 controls were enrolled in the study. Carriers of CHIP had a risk of CAD that was 1.9 times higher in comparison with noncarriers (95% CI: 1.4–2.7), and a risk of MI 4 times higher than noncarriers (95% CI: 2.4–6.7). Mutations in DNMT3A, TET2, ASXL1 and JAK2 were associated with CAD. The authors concluded that somatic mutations in hematopoietic cells are implicated in the development of atherosclerosis [45]. Although the relationship between CHIP and MGUS remains to be clarified [47], both entities may co-exist, especially in elderly patients. However, the exact genetic abnormalities contributing to the increased risk of CVD in patients with MGUS, including those without CHIP, have to be elucidated in future research.

### 3.2. Vascular Calcification

Vascular calcification is a pathological condition characterized by the deposition of hydroxyapatite in the extracellular matrix at the intima and medial of the arteries [48,49]. Intimal calcification is present in atherosclerosis, whereas medial arterial calcification occurs irrespective of atherosclerosis, is an independent predictor of cardiovascular mortality and is commonly seen in patients with DM and chronic kidney disease [48,49]. The interaction of RANKL with its signaling receptor RANK and osteoprotogerin is implicated in the pathogenesis of the vascular calcification [50]. More specifically, activation of RANKL in vascular smooth muscle cells leads to calcification [50]. Osteoprotogerin plays an important role in bone metabolism as a decoy receptor for RANKL in the RANK/RANKL/osteoprotogerin axis, effectively limiting the action of RANKL, whereas this signaling cascade plays a key role in myeloma bone disease [50,51]. Serum levels of RANKL and RANKL/OPG have been found to be higher in patients with MGUS than in controls [52], especially in patients with MGUS at high risk for progression to symptomatic MM [53]. Therefore, biomarkers related to bone metabolism are increased in patients with MGUS, whereas they may also promote vascular calcification and, subsequently, may lead to an increased risk for CVD events.

### 3.3. AL Amyloidosis

Light chain AL amyloidosis is an MGUS-associated condition with a severe impact on the cardiovascular system due to the deposition of monoclonal light chains. The accumulation of immunoglobulin light chain-based amyloid fibrils in cardiac tissue can cause restrictive cardiomyopathy [54,55]. Amyloid deposits infiltrate the myocardium, impairing normal cardiac function and thickening the walls of the heart while also weakening their contractility, ultimately resulting in heart failure. Cardiac biomarkers indicative of heart injury (e.g., NTproBNP) constitute key prognostic factors both at the diagnosis and at relapse after prior treatment with proteasome inhibitors (e.g., bortezomib) and/or anti-CD38 monoclonal antibodies (e.g., daratumumab) [56,57,58,59,60]. Amyloid deposits may also lead to arrhythmias, due to structural disruptions in the cardiac conduction system. The infiltration of amyloid fibrils into the walls of vessels may also contribute to the development of atherosclerosis, further elevating the risk of CV events. Thromboembolic events are also frequently reported among patients with AL amyloidosis [61]. Although this may be partially attributed to the increased incidence of atrial fibrillation and development of intracardiac thrombi, it may be also associated with a systemic vascular frailty status in combination with the presence of CV comorbidities [62]. Furthermore, additional organs, such as the kidneys, may be impacted by AL amyloidosis, which would increase the stress on the CV system [55]. In order to manage the cardiovascular issues associated with AL amyloidosis, prompt detection and management are essential [63]. This is because treating the underlying amyloid deposition before causing irreversible tissue damage may reduce the risk for CV complications and improve patient prognosis [64].

### 3.4. Cryoproteins

#### 3.4.1. Cryoglobulinemia

Cryoglobulinemia is a syndrome characterized by the presence of serum immunoglobulins that precipitate at cold temperatures (<37 °C) and re-dissolve at 37 °C when rewarmed [65]. Cryoglobulinemia is classified according to the Brouet classification into three categories based on Ig composition. Type I cryoglobulinemia (monoclonal Igs, typically IgG or IgM rarely IgA or free light chain) develops in the setting of lymphoproliferative or hematologic disorders of B cell lineage; type II mixed cryoglobulinemia [monoclonal IgM (or IgG or IgA) with rheumatoid factor activity and polyclonal Ig] develops in infections, autoimmune diseases and lymphoproliferative disorders; type III cryoglobulinemia (polyclonal IgG (all isotypes) and polyclonal IgM) is associated with autoimmune disorders and infections [65,66]. Type I cryoglobulinemia is commonly observed in patients with MGUS. In a study by Sidana et al. of 102 people diagnosed with type I cryoglobulinemia, 39 (38%) had underlying MGUS [67].

The most common manifestations of cryoglobulinemia are cutaneous, musculoskeletal, peripheral nerve, kidney and pulmonary involvement. Cryoglobulinemia may also lead to thrombosis and cardiovascular events. In a study by He et al. of 108 patients with cryoglobulinemia who were admitted to the hospital, seven (6.8%) had cardiac involvement [68]. Six patients had enlarged cardiac chambers, including left ventricular enlargement, with left and right ventricular enlargement and left atrial enlargement found in two subjects; five participants had reduced left ventricular systolic motion and ejection fraction; three had pericardial effusion; and one had severe aortic insufficiency [68]. The mechanisms that lead to cardiovascular events include small vessel vasculitis (cryoglobulinemic vasculitis), vascular occlusion by the cryoprecipitate, thrombotic microangiopathy and hyperviscosity syndrome [9,66].

#### 3.4.2. Cold Agglutinin Disease

Cold agglutinin disease is an autoimmune hemolytic anemia that is either primary (idiopathic) or secondary to conditions such as infections, autoimmune disease and B cell lymphoproliferative disorders [69]. Primary cold agglutinin disease is mainly characterized by clonal expansion of kappa-positive B cells in the bone marrow and a monoclonal immunoglobulin M (IgM)-kappa paraprotein [3,4]. Broome et al. performed a retrospective study to examine the risk of thrombotic events in patients with cold agglutinin disease [70]. Overall, 608 patients with CAD and 5873 matched controls were enrolled in the study. The study showed that those with cold agglutin disease had more often venous, arterial and cerebral thrombotic events than controls [adjusted HR (95% CI): 1.94 (1.64–2.30)]. Regarding arterial thromboses, those with cold agglutin disease had an increased risk of arterial embolism and thrombosis as well as MI (HR (95% CI): 1.93 (1.37–2.72)) and had an increased risk for cerebral events including cerebral infarction, occlusion and stenosis of cerebral and precerebral arteries and vascular syndromes of the brain in cerebrovascular diseases, transient cerebral ischemic attacks, and related syndromes (adjusted HR (95% CI): 1.26 (1.00–1.60)) [70].

### 3.5. Autoimmunity in MGUS

Patients with antiphospholipid syndrome have an increased risk for arterial and venous thromboses, and recommendations for cardiovascular risk management have been published by The European Alliance of Associations for Rheumatology (EULAR) [71,72]. Monoclonal gammopathies, including Waldenstrom disease, multiple myeloma and MGUS have been associated with the presence of antiphospholipid antibodies [73,74,75,76,77]. In a study of 93 participants, those with MGUS had a higher incidence of serum antiphospholipid antibodies than healthy controls [76]. In another study by Doyle et al., nine subjects were identified as having thrombotic antiphospholipid syndrome with associated monoclonal gammopathy [78]. The rate of thrombosis recurrence in all participants with monoclonal gammopathy was 7/9 (89%) versus 15/36 (42%) in those without monoclonal gammopathy (*p* = 0.058). The authors suggested that the co-presence of antiphospholipid syndrome in patients with monoclonal gammopathy is a mechanism that may contribute to recurrent thrombosis [78].

### 3.6. Other Factors

Case reports have shown that MGUS is associated with immunoglobulin-mediated vasculitis [79] and immunoglobulin-mediated leukocytoclastic vasculitis [80]. This may be related to paraprotein-induced immune complex deposition and underlying inflammatory processes leading to occlusion and cardiovascular events. In addition, monoclonal paraproteins have been associated with impaired platelet function, which may lead to platelet hyperreactivity and thrombotic events [81]. Moreover, in vitro studies have shown that prothrombotic coagulation abnormalities are present in MGUS patients. Auwerda et al. found an increase in factor VIII and von Willebrand factor in subjects with MGUS and systemic amyloidosis, which was similar to the increase observed in patients with untreated multiple myeloma [82]. Similarly, Crowley et al. reported that among 24 people (8 with MGUS, 8 with multiple myeloma and 8 healthy controls), people with MGUS have a distinct coagulation profile, which is intermediate between patients with myeloma and normal controls [83].

## 4. Future Perspectives

Multiple myeloma is associated either directly or indirectly with anti-myeloma drugs with increased cardiovascular risk and recommendations regarding the management of cardiovascular risk have been published [8]. Similar to multiple myeloma, recent studies have demonstrated that MGUS may be associated with CVD [10,11]. However, since the median age at diagnosis is 65–70 years, patients also have several other cardiovascular risk factors, such as hypertension, dyslipidemia, obesity and type 2 DM. Hence, further prospective studies are needed to examine whether MGUS is an independent risk factor of cardiovascular events. In addition, population studies are needed to examine the prevalence of these established cardiovascular risk factors among people with MGUS and compare them to the general population. If cardiovascular risk factors are more common in people with MGUS, then more intense management may be needed in terms of blood pressure, cholesterol levels monitoring, glycemic control, weight management and smoking cessation. Moreover, apart from studies using hard cardiovascular endpoints such as cardiovascular death, MI and stroke, studies examining whether MGUS is associated with subclinical atherosclerosis using markers such as CT calcium score, intravascular ultrasonography, magnetic resonance imaging, pulse wave velocity and carotid intima-media thickness are needed to elucidate whether MGUS is associated with high atherosclerosis burden.

## 5. Conclusions

MGUS is a premalignant plasma cell disorder with a 1% annual rate of progress to multiple myeloma. MGRS is a well-established condition indicative of treatment in patients with MGUS, and lately, studies have shown that MGUS may be associated with other comorbidities, such as arterial and venous thrombotic events. Herein, we show that MGUS is associated with increased CVD, and people with MGUS and cardiovascular events have worse outcomes than people without MGUS. Therefore, people with MGUS may benefit from regular cardiovascular risk assessment and management as well as close hematological follow-up to monitor disease progression. We advocate for strict adherence to the established guidelines [12] for cardiovascular disease prevention, risk stratification, risk-adapted follow-up, and prompt intervention in order to decrease cardiovascular morbidity and mortality in patients with MGUS.

## Data Availability

Data available on request from the corresponding author.
